# The Mediator Role of Body Image-Related Cognitive Fusion in the Relationship between Disease Severity Perception, Acceptance and Psoriasis Disability

**DOI:** 10.3390/bs10090142

**Published:** 2020-09-19

**Authors:** Vera Almeida, Ângela Leite, Diana Constante, Rita Correia, Isabel Filipa Almeida, Maribel Teixeira, Diogo Guedes Vidal, Hélder Fernando Pedrosa e Sousa, Maria Alzira Pimenta Dinis, Ana Teixeira

**Affiliations:** 1 CESPU, Institute of Research and Advanced Training in Health Sciences and Technologies, Rua Central de Gandra 1317, 4585-116 Gandra PRD, Portugal; vera.almeida@iucs.cespu.pt (V.A.); a21873@alunos.cespu.pt (D.C.); ritacorreia96psic@gmail.com (R.C.); maribel.teixeira@iucs.cespu.pt (M.T.); ana.teixeira@iucs.cespu.pt (A.T.); 2 Faculty of Philosophy and Social Sciences, Portuguese Catholic University, Rua de Camões 60, 4710-362 Braga, Portugal; 3 UCIBIO/REQUIMTE, MedTech-Laboratory of Pharmaceutical Technology, Department of Drug Sciences, Faculty of Pharmacy, University of Porto, Rua de Jorge Viterbo Ferreira 228, 4050-313 Porto, Portugal; ifalmeida@ff.up.pt; 4 UFP Energy, Environment and Health Research Unit (FP-ENAS), University Fernando Pessoa (UFP), Praça 9 de Abril 349, 4249-004 Porto, Portugal; diogovidal@ufp.edu.pt; 5 Department of Mathematics (DM. UTAD), University of Trás-os-Montes and Alto Douro, Quinta de Prados, 5001-801 Vila Real, Portugal; hfps@utad.pt

**Keywords:** acceptance, body image-related cognitive fusion, disease severity perception, psoriasis disability, self-compassion

## Abstract

Psoriasis is a long-term skin disorder without a cure, whose patients are particularly susceptible to mental health diseases. Using a sample of patients diagnosed with psoriasis, this study aimed to: (1) identify the clinical and positive psychological variables that contribute the most to psoriasis disability and (2) assess the mediator role of body image-related cognitive fusion in the relation between disease severity perception and acceptance and self-compassion, on one hand, and psoriasis disability on the other. This is an initial cross-sectional exploratory study, with 75 patients diagnosed with psoriasis (males 52%; mean age 54.99 ± 13.72) answering a sociodemographic and a clinical questionnaire, the Psoriasis Disability Index (PDI), the Cognitive Fusion Questionnaire—Body Image (CFQ-BI), the Acceptance and Action Questionnaire—II (AAQ-II), and the Self-Compassion Scale (SCS). Descriptive and inferential statistics were used to characterize and assess the measures and the final model used. Through path analysis and a hierarchical multiple linear regression, it was found that the variables that significantly contributed to psoriasis disability were years of education, impact on social life and body image, explaining 70% of the variance. Body image-related cognitive fusion was a significant mediator in the relationship between disease severity and acceptance, and psoriasis disability. The implications of this study are considered to be extremely relevant, since it will allow additional information to be provided to psoriasis patients, appropriated to their educational level, aiming to reduce distorted perceptions of disease severity and intervene in the ability to accept this specific and important chronic health condition.

## 1. Introduction

Psoriasis is a long-term skin disorder that has no cure [1] and may express itself as an active severe disease or as mild stable disease [2]. Psoriasis patients are more susceptible to mental health diseases than patients with other dermatological conditions [3]. It is recognized that the risk of depression, anxiety, and suicidality is higher in patients with psoriasis [4]. The impact of psoriasis on patients’ lives can be assessed by different instruments, namely, the Psoriasis Disability Index (PDI) [5]. However, the variables that contribute to the impact of this specific disease on mental health are not fully known.

Several psychological constructs have been studied in clinical samples of patients with psoriasis, e.g., depression [6], anxiety [7], embitterment [8], stigmatization [9], isolation [10], quality of life [11] and body image [12,13]. All these studies report worse outcomes for patients with psoriasis than for controls, i.e., more depression, more anxiety, more embitterment, more stigmatization, more isolation, less quality of life and worse body image.

Other studies have focused on positive constructs, such as acceptance [14] and self-compassion [15]. These studies assume that acceptance and self-compassion improve the perception of the disease, thus contributing to a better quality of life; acceptance is recognized as the ability to experience undesired private events, in order to pursue one’s values and goals [16]. On the contrary, experiential avoidance is the effort to change the form, frequency, or situational sensitivity of sensible private events, namely, thoughts, feelings and physiological sensations, even if this leads to inconsistent actions involving values and goals [16,17]. Acceptance and experiential avoidance are examples of psychological flexibility and inflexibility, respectively. Psychological flexibility is the “ability to fully contact the present moment and the thoughts and feelings it contains without needless defense” [18] (p. 7). Psychological inflexibility includes rigid dominance of psychological reactions in guiding actions. This happens when people fuse with evaluative and self-descriptive thoughts and try to avoid experiencing undesired internal events, decreasing their contact with the present moment [17]. Self-compassion is a complex construct including three essential elements: treating oneself with kindness, recognizing one’s imperfections as part of the common human experience, and maintaining a balanced awareness of one’s thoughts and feelings [19].

In a state of cognitive fusion, persons behave according to their thoughts, considering them real, truthful and wise; cognitive fusion is the degree to which an individual interacts with events regarding their verbal functions rather than their direct ones [20]. This leads to self-identification with one’s thoughts and inabilities, considering them as part of an inner experience [21]. Body image is a concept that includes the subjective perceptions of the individual formed by their feelings and beliefs related to his/her own body [22]. Body image-related cognitive fusion is linked to unfavorable social rank perceptions [23]. Trindade and Ferreira [23] found that women “who get fused and tangled with thoughts relating to body image have greater tendencies of developing disordered mechanisms in order to “correct” their body dissatisfaction and social disadvantage” (p. 72). Body image dissatisfaction can be due to the perception of a significant discrepancy between one’s real body image and one’s desired one [24]. Since psoriasis manifests itself with the appearance of physical lesions, a negative change in the patient’s body image may lead to a negative psychosocial impact, resulting in an increase in body coverage as well as inhibition and reduction of physical exposure [25]. Depending on the visibility, appearance and location, psoriasis physical lesions may be stigmatizing for patients, leading to low self-esteem and negative perception of their body image [26]. Łakuta and Przybyła-Basista [27] found that attitude towards the body mediates the relationship between the severity of psoriasis and depression, and Lawrence, Fauerbach and Thombs [28] found that the importance of appearance moderates the relationship between the subjective burn scars severity and body-esteem.

However, the mediator role of body image-related cognitive fusion between positive psychological constructs and psoriasis disability has not yet been studied. To fill this gap, this study aims to (1) identify the clinical and positive psychological variables that contribute the most to quality of life (psoriasis disability) and (2) assess the mediator role of body image-related cognitive fusion in the relationship between disease severity perception, acceptance and self-compassion, on one hand, and psoriasis disability on the other, in a sample of patients diagnosed with psoriasis.

## 2. Materials and Methods

### 2.1. Participants and Procedures

The sample selection of 75 patients diagnosed with psoriasis (mean age 54.99 ± 13.72) intended to be representative of the Portuguese population, comprising participants from all regions of the country and with different professions. In line with this, participants were recruited by the Portuguese Psoriasis Association (PSO Portugal). According to the PSO Portugal, psoriasis affects 1 to 3% of the Portuguese population [29]. In this initial exploratory study, the inclusion criteria were to be 18 years old, educated (i.e., at least 4 years of education) and with a diagnosis of psoriasis. All procedures performed in studies involving human participants were in accordance with the ethical standards of the institutional research committee under whose responsibility the study was carried out (31/CE-IUCS/2019) and with the 1964 Helsinki declaration and its later amendments. In order to guarantee the ethical aspects involved, the objectives of the study and the voluntary and confidential nature of the participation were explained to participants. Informed consent, its acceptance and fulfillment were mandatory.

### 2.2. Measures

#### 2.2.1. Sociodemographic and Clinical Questionnaire

The *sociodemographic questionnaire* included questions related to age, gender, marital status, education and occupation, and the *clinical questionnaire* contained questions related to year of diagnosis of psoriasis, family history of psoriasis, existence of other diseases, satisfaction with current psoriasis treatment, disease severity perception, taking anxiolytics and antidepressants, and impact of psoriasis disease on social life.

#### 2.2.2. Psoriasis Disability Index

The *Psoriasis Disability Index* (PDI) [5] assesses the impact of psoriasis on patients’ lives. It consists of 15 items divided in 5 subscales: daily activities, work/school or alternative questions, personal relationships, leisure activities and treatment. The score of each question is given in 4 possible answers: nothing (0), slightly (1), quite (2) and very (3), with a minimum of 0 points and a maximum of 45 points. A higher value corresponds to a higher score of disability. The Portuguese version was validated by Fernandes et al. [30], with a good level of internal consistency (α = 0.89).

#### 2.2.3. Cognitive Fusion Questionnaire—Body Image

The Cognitive Fusion Questionnaire—Body Image (CFQ-BI) [31] assesses the extent to which individuals “merge” with their own body image. It consists of 10 items on a Likert scale of 7 points, where 1 corresponds to “never true” and 7 to “always true” for a total of 70 points. The Portuguese version was translated and validated by Ferreira et al. [24], with a very good internal consistency (α = 0.96), as well as convergent, divergent and temporal.

#### 2.2.4. Acceptance and Action Questionnaire—II

*Acceptance and Action Questionnaire—II* (AAQ-II) [18] assesses psychological flexibility and experiential avoidance. It consists of a scale of 10 Likert-type items ranging from 1 “never true” to 7 “always true” for a total of 49 points. The quotation is obtained through the sum of scores, with higher scores indicating greater psychological inflexibility or avoidance. The Portuguese version was translated and adapted by Pinto-Gouveia and colleagues [32], with a very good level of internal consistency (α = 0.90).

#### 2.2.5. Self-Compassion Scale

The *Self-Compassion Scale* (SCS) [33] assesses the self-compassion through 26 items and aims to measure the three basic components of self-compassion: self-kindness, common humanity and mindfulness. It is divided into 6 subscales: self-kindness, self-judgment, common humanity, isolation, mindfulness and over-identification. The items are quoted on a 5-point Likert scale, where 1 corresponds to “almost never” and 5 to “almost always” for a total of 130 points. The quotation is made by summing the score of all items. Higher scores mean more self-compassion. The Portuguese version has been translated and validated by Castilho and colleagues [34], with very good internal consistency (α = 0.92).

### 2.3. Data Analysis

The sample was calculated a *priori* with version 4.0 of the Free Statistics Calculators, respecting the criteria required for different types of studies, with a medium effect size (*d* = 0.50) and an alpha of 0.05, specifying the minimum sample size needed to detect the adjusted effect according to the structural complexity of the model (*n* = 63). However, 75 participants were recruited through PSO Portugal and thus included in the study. Descriptive statistics were used to characterize the sample from a sociodemographic and clinical point of view, as well as the psychological variables. Through a Student’s *t*-test, the differences between the values of psychological variables obtained in the study and those obtained in studies conducted with the original versions of the instruments used in this study, were calculated. Chronbach’s alpha was used to assess the reliability of the measures. A Pearson correlation was performed to analyze the associations between demographic, clinical and psychological variables with the outcome (PDI). In order to examine the comparative strength of direct and indirect effects among variables, a path analysis was chosen. The robust maximum likelihood estimation procedure was used to account for the non-normality of the data. The adequacy of the model was assessed according to the goodness-of-fit indexes. The Satorra-Bentler Scale chi-square test was used. Non-significance of *p* value (*p* > 0.05) and the ratio (S-B*χ*^2^)/*df* < 3 represent a good model fit. Since the significance of a chi-square test is dependent on the number of participants, other goodness-of-fit indexes were used, namely, the Comparative Fit Index (CFI), with a maximum value of 1.00, for which results above 0.90 suggest a good fit; and the Root Mean Square Error Approximation (RMSEA), for which values below 0.05 indicate a good fit, and values up to 0.08 are acceptable. Additionally, a hierarchical multiple linear regression was performed to assess the variables that significantly contributed to psoriasis disability. In this study, there were no missing data. All analyses were conducted with IBM SPSS and AMOS version 25, and a 0.05 level of significance was considered.

## 3. Results

### 3.1. Sample Characterization

The total sample (*n* = 75) used in this exploratory study is mostly male, with a mean age of 54.99 years, married or committed, not graduated and active (working). The sample reports having a clinical diagnosis of psoriasis, on average, for 23.96 years. Most of the sample is satisfied with the current treatment, has no other diseases, and does not take anxiolytics and antidepressants. Additionally, the disease does not have a great impact in their social life, and the family has not history of psoriasis. Detailed sociodemographic and clinical data is presented in Table 1.

### 3.2. Comparison Between Psoriasis Disability Index, Body Image-Related Cognitive Fusion, Acceptance and Action, and Self-Compassion Frequencies in this Study and in the Original Authors’ Studies

There are statistically significant differences in most of the comparisons made between the values presented in this study and the values presented by the original authors’ instruments [24,30,32,34] with clinical samples in their studies. The sample of this study shows higher values in all dimensions of the PDI, including the total, except in the *work or school* dimension (Table 2). The same can be stated for the self-compassion scale, except for the *self-judgment, isolation* and *over-identification* dimensions. With respect to body image-related cognitive fusion and acceptance, the sample of this study presents lower values, although very close to the last one.

### 3.3. Correlations between Sociodemographic, Clinical and Psychological Variables

From Table 3, only the significant correlations stand out (the bold values indicated in Table 3). Significant correlations are presented between sociodemographic and clinical variables with psychological variables. Finally, the correlations between psychological variables stand out. Years of education correlates negatively with treatment adherence (PDI). The existence of other diseases in addition to psoriasis correlates positively with acceptance and negatively with self-compassion, self-kindness, self-judgment and isolation. Family history of psoriasis correlates positively with common humanity. Disease severity perception correlates positively with PDI (total), leisure and body image-related cognitive fusion and negatively with self-kindness. Satisfaction with treatment correlates negatively with work or school (PDI). Taking anxiolytics and or antidepressants correlates positively with personal relationships, leisure and acceptance and negatively with self-compassion, self-judgment, isolation and over-identification. Impact in social life correlates positively with PDI (total), daily activities, work or school, personal relationships, leisure, body image-related cognitive fusion and acceptance and negatively with self-compassion, self-judgment, isolation and over-identification. Self-compassion correlates negatively with PDI (total) and all its dimensions, except treatment, body image-related cognitive fusion and acceptance. Acceptance correlates positively with PDI (total) and all its dimensions, except treatment. Body image-related cognitive fusion correlates positively with PDI (total) and all its dimensions, except treatment, as shown in the correlations between variables presented in Table 3.

### 3.4. Hierarchical Multiple Linear Regression: Predictors of Psoriasis Disability Index (Final Model)

In Table 4, model 1 includes sociodemographic variables. In addition to sociodemographic variables, model 2 in Table 4 also includes clinical variables. Finally, model 3 presented in Table 4 includes sociodemographic, clinical and psychological variables. In relation to hierarchical multiple linear regression, the variables that significantly contribute to PDI are years of education, impact on social life and body image (model 3 in Table 4), explaining 70% of the variance [*F* (10, 65) = 14.794; *p* < 0.001].

### 3.5. Body Image as Mediator of Acceptance and Disease Severity Perception, and PDI

**Figure 1:**
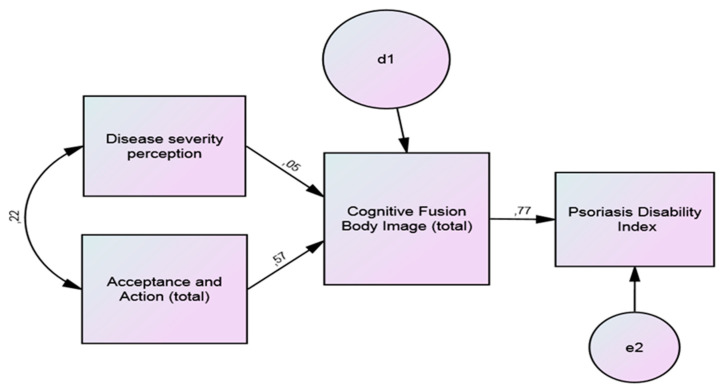
Path analysis of body image as mediator of acceptance and disease severity perception, and Psoriasis Disability Index (PDI); fit indices: *χ*^2^ = 1.28, *p* < 0.001, CFI = 0.991, RMSEA = 0.053.

The results of the path analysis with the standardized regression coefficients for PDI are presented in Figure 1, and this model has a good fit. d1 and e2 are errors, i.e., the proportion of the variance that is not explained by the variable.

Body image-related cognitive fusion was found to have a significant mediator role in the relationship between disease severity and acceptance on one hand, and the PDI on the other, being the indirect effects significative (Table 5).

## 4. Discussion

The aims of this study were to identify the variables that contribute most to the quality of life of psoriasis patients (psoriasis disability) and to assess the mediator role of body image-related cognitive fusion in the relationship between disease severity perception, acceptance, self-compassion and psoriasis disability.

In relation to the representativeness of the sample, it was found that 1% of individuals with Psoriasis in Portugal are represented, which is in accordance with the latest statistics available [29]. Alongside, and in agreement with the available data [29], men and women in this study have the same chance to develop psoriasis and are more likely to be diagnosed between 20 and 30 years old. Although psoriasis is usually associated with somatic comorbidities, with cardiovascular risk and associated diseases being one of its aspects, the sample in this study mainly reports no other diseases and does not take anxiolytics and antidepressants. This discrepancy can be explained by the fact that most of the sample perceives the severity of psoriasis disease as moderate.

Self-compassion did not contribute to explaining psoriasis disability, although Stuntzner [35] considers that compassion is a component of adjustment to disability. Regarding the first objective of the study, the clinical and positive psychological variables that contribute most to quality of life (psoriasis disability), years of education, impact of psoriasis on social life and body image-related cognitive fusion were identified as contributors in explaining psoriasis disability. In this study, the variable years of education is negatively correlated with treatment (PDI), suggesting that patients with more education deal with and manage the disease better than those with less education. According to Groot and colleagues [36], maternal educational attainment and equivalized household income were inversely associated with psoriasis in offspring. Löfvendahl and colleagues [37] found that fewer years of education in patients with psoriasis was associated with increased use of primary health services. However, Kimball and colleagues [38] found a positive association between poor psoriasis control and lower education.

Although most of the sample reports that the disease has little impact on their social life, this impact is one of the variables that significantly contributes to explaining the quality of life (psoriasis disability) of the patients. In fact, physical expression of the disease causes embarrassment in the patients, leading them to avoid social exposure and promoting isolation behaviors, which, in turn, impact the quality of life associated with psoriasis [39]. Indeed, patients with psoriasis and their families have psychosocial impairments [40]. Psoriasis diminishes the life potential of affected patients, a phenomenon called “Cumulative Life Course Impairment” [41]: stigmatization, psychological morbidity and social isolation may, over time, deeply impact education, profession, life course and income [42].

Additionally, body image-related cognitive fusion significantly explains PDI, correlating positively with it. The visible appearance of the physical lesions negatively impacts body image-related cognitive fusion, which leads to a decrease in patient’s quality of life [43]. Wojtyna et al. [44] found that improving the body image of patients with psoriasis by reducing its salience in their personal lives may prevent depression, above all, in women. However, Trindade and Ferreira [23] proposed the promotion of cognitive fusion and acceptance of inner events to construct a healthy skepticism about one’s thoughts.

In relation to the second objective of the study, the mediator role of body image-related cognitive fusion between disease severity perception and acceptance and quality of life (psoriasis disability) was confirmed. This result is in line with Łakuta and Przybyła-Basista’s [45] study, which found that emotional attitude towards the body mediates the relationship between self-reported severity of psoriasis and depression. It is also in line with Lawrence and colleagues’ [28] study, which found that the importance of appearance moderated the relationship between subjective burn scars severity and body-esteem.

Although psychosocial comorbidities are not always proportional to psoriasis severity and perception of psoriasis severity is not always proportional to psoriasis severity [45], patients with psoriasis report more subjective health complains than controls [46]. The location and size of the physical lesions is the second most important factor that contributes to explaining psoriasis disease severity perception [47], suggesting a relationship between disease severity perception and body image that is negatively affected in patients with psoriasis [44]. In addition, body image-related cognitive fusion is positively associated with anxiety and depression [31,48].

Acceptance of the disease is a strong determinant of quality of life in psoriasis patients [49]. It is associated with body image-related cognitive fusion, which has a buffering effect on the consequences of psoriasis [49]. Perceiving the disease as an obstacle and being aware that the health locus of control is internal determines the degree of acceptance of psoriasis [14]. Acceptance of the disease and satisfaction with the body and its positive image are associated with feelings of personal happiness [13]. In fact, Acceptance and Commitment Therapy (ACT) seeks to counteract cognitive fusion and experiential avoidance, which are two processes of psychological inflexibility [50]. Also, the aggregated effect of experiential avoidance and cognitive fusion is relevant to perceived burdensomeness in the extreme form of experiential avoidance, i.e., suicide [51].

## 5. Conclusions

Psoriasis is a specific and important chronic health condition, a long-term skin disorder with no cure, and diagnosed patients are particularly susceptible to experience worse quality of life.

Using path analysis and hierarchical multiple linear regression, the results indicate that the variables that significantly contribute to psoriasis disability are years of education, impact on social life and body image. Body image-related cognitive fusion is a significant mediator in the relationship between disease severity and acceptance, and psoriasis disability.

The main findings and strengths of this study are important on three different levels: (1) providing psoriasis patients with information appropriate to their educational level in order to reduce distorted perceptions of disease severity that may compromise their quality of life and adaptation to the disease, (2) intervening in the ability to accept this specific long-term chronic health condition, and (3) addressing issues associated with a negatively disproportionate body image in relation to the extent and characteristics of the physical lesions.

The limitations of this study are related to the reduced number of samples used in this initial exploratory study and the nature of the instruments, which are all self-reporting. In the future, studies with a larger sample and a longitudinal design will be able to confirm and strengthen the results obtained, contributing to a better understanding of the psoriasis disease experience and enabling a multidisciplinary approach within integrated psoriasis treatment.

## Figures and Tables

**Table 1:** Sociodemographic and clinical characterization of the sample (*n* = 75).

*Sociodemographic Variables*	%
*Age (M ± SD)*	54.99 ± 13.72
*Gender (%)*	
Female	48.0
Male	**52.0**
*Marital Status (%)*	
Not married/Committed	24.0
Married/Committed	**76.0**
*Education (%)*	
Non-graduate	**53.3**
Graduate	46.7
*Occupation (%)*	
Active	**57.3**
Inactive	42.7
*Clinical variables*	
*Years of diagnosis (M ± SD)*	23.96 ± 15.40
*Family history of psoriasis (%)*	
No	**57.3**
Yes	42.7
*Other diseases (%)*	
No	**56.0**
Yes	44.0
*Satisfaction with current treatment (%)*	
No	30.7
Yes	**69.3**
*Disease severity perception (%)*	
Mild	22.7
Moderate	**58.7**
Severe	18.7
*Anxiolytics and antidepressants (%)*	
No	**73.3**
Yes	26.7
*Impact in social life (%)*	
None or little	**68.0**
Some or a lot	32.0

*M* = mean; *SD* = Standard deviation; % = percentage; In bold the higher percentages.

**Table 2:** The Psoriasis Disability Index, Body Image-Related Cognitive Fusion, Acceptance and Action and Self-Compassion Frequencies (*n* = 75) and a comparison of original authors’ instruments with clinical samples.

Variables	This Study				Original Authors’ Instruments Studies [24,30,32,34]				Statistical Comparison			
	*n*	*M*	*SD*	α	*n*	*M*	*SD*	*α*	*t*	*df*	*p*	*d*
*Psoriasis Disability Index (total)*	75	8.44	7.33	0.82	171	2.10	1.60	0.89	7.414	77, 111	**<0.001**	1.95
*Daily activities*	75	4.44	3.95	0.87	171	3.10	2.20	0.83	2.756	94, 743	**0.007**	0.42
*Work or school*	75	0.93	1.48	0.56	171	0.99	1.70	0.75	−0.279	160, 962	0.780	0.04
*Personal relationships*	75	0.71	1.10	0.80	171	1.40	2.20	0.87	−3.273	239, 942	**0.001**	0.40
*Leisure*	75	1.67	1.88	0.57	171	1.70	2.10	0.85	−0.111	156, 724	0.912	0.02
*Treatment (1 item)*	75	0.69	0.77	−	171	2.30	2.90	−	−6.738	216, 210	**<0.001**	0.76
*Body Image Cognitive Fusion (total)*	75	28.15	13.89	0.98	31	51.74	12.12	0.96	8.725	63, 792	**<0.001**	1.81
*Acceptance and Action (total)*	75	33.19	8.21	0.73	297	33.47	9.46	0.90	0.256	128, 332	0.799	0.03
*Self-Compassion (total)*	75	84.51	15.83	0.83	298	60.25	15.79	0.92	−12.643	89, 803	**<0.001**	1.53
*Self-Kindness*	75	13.77	4.35	0.83	298	10.57	3.87	0.92	−6.234	83, 743	**<0.001**	0.78
*Self-Judgment*	75	17.29	4.31	0.86	298	18.41	4.05	0.93	2.174	84, 904	**0.032**	0.27
*Common Humanity*	75	12.19	3.45	0.80	298	10.09	3.15	0.85	−5.102	84, 277	**<0.001**	0.64
*Isolation*	75	14.17	3.33	0.84	298	14.77	3.31	0.89	1.502	86, 246	0.137	0.18
*Mindfulness*	75	12.85	3.19	0.73	298	9.55	2.88	0.85	−8.678	84, 041	**<0.001**	1.09
*Over-identification*	75	14.23	3.19	0.79	298	14.77	3.24	0.88	1.408	86, 805	0.163	0.17

*n* = frequencies; *M* = mean; *SD* = standard deviation; *α* = Cronbach’s alpha; *t* = Student’s *t*-test; *df* = degrees of freedom; *p* = *p* value; *d* = Cohen’s *d* effect size; **bold** = statistically significant differences.

**Table 3:** Correlations between sociodemographic, clinical and psychological variables.

	1	2	3	4	5	6	7	8	9	10	11	12	13	14	15	16	17	18	19	20	21	22
1. Years of education	1	−0.028	0.053	−0.004	0.070	0.106	−0.031	−0.168	−0.187	−0.085	−0.044	−0.070	−0.236 *	−0.067	0.111	−0.079	0.067	−0.205	0.012	−0.055	−0.089	−0.076
2. Other diseases		1	0.159	0.097	0.182	0.194	0.098	0.087	0.120	−0.088	0.066	0.115	0.004	0.181	0.260 *	−0.292 *	−0.239 *	−.230 *	−0.189	−0.330 **	−0.120	−0.140
3. Family history of psoriasis			1	0.054	0.106	0.089	−0.022	0.096	0.137	−0.016	−0.015	0.110	−0.007	0.116	0.106	0.042	0.132	−0.072	0.268 *	−0.086	0.083	−0.155
4. Disease severity perception				1	0.004	0.273 *	0.460 **	0.247 *	0.213	0.195	0.173	0.311 **	−0.134	0.269 *	0.218	−0.215	−0.229 *	−0.185	−0.081	−0.122	−0.101	−0.186
5. Satisfaction with treatment					1	0.009	−0.051	−0.047	−0.036	−0.246 *	−0.020	0.021	0.187	−0.148	−0.031	−0.017	0.085	−0.029	0.045	−0.096	−0.040	−0.071
6. Anxiolytics/antidepressants						1	0.221	0.200	0.163	0.048	0.272 *	0.252 *	−0.034	0.219	0.252 *	−0.309 **	−0.219	−.330 **	0.002	−.286 *	−0.210	−0.281 *
7. Impact in social life							1	0.687 **	0.671 **	0.462 **	0.494 **	0.564 **	0.114	0.645 **	0.432 **	−0.329 **	−0.218	−0.432 **	0.026	−0.319 **	−0.052	−0.394 **
8. PDI (total)								1	0.938 **	0.656 **	0.677 **	0.822 **	0.455 **	0.764 **	0.521 **	−0.491 **	−0.353 **	−0.504 **	−0.058	−0.440 **	−0.242 *	−0.510 **
9. Daily activities									1	0.492 **	0.537 **	0.655 **	0.475 **	0.756 **	0.481 **	−0.447 **	−0.343 **	−0.437 **	−0.065	−0.379 **	**−0**.238 *	−0.458 **
10. Work or school										1	0.344 **	0.495 **	0.088	0.466 **	0.423 **	−0.364 **	−0.218	−0.370 **	−0.042	−0.356 **	−0.191	−0.403 **
11. Personal relationships											1	0.591 **	0.148	0.572 **	0.410 **	−0.439 **	−0.293 *	−0.495 **	−0.078	−0.373 **	−0.217	−0.420 **
12. Leisure												1	0.208	0.644 **	0.408**	−0.411 **	−0.312 **	−0.436 **	−0.071	−0.354 **	−0.175	−0.406 **
13. Treatment													1	0.100	0.088	−0.039	0.003	−0.062	0.144	−0.158	0.025	−0.131
14. Body image (total)														1	0.540 **	−0.615 **	−0.417 **	−0.570 **	−0.194	−0.532 **	−0.341 **	−0.607 **
15. Acceptance Action (total)															1	−0.590 **	−0.264 *	−0.540 **	−0.172	−0.625 **	−0.347 **	−0.654 **
16. Self-compassion (total)																1	0.758 **	0.732 **	0.570 **	0.770 **	0.752 **	0.771 **
17. Self-kindness																	1	0.292 *	0.657 **	0.261 *	0.737 **	0.282 *
18. Self-judgment																		1	0.034	0.773 **	0.214	0.825 **
19. Common Humanity																			1	0.133	0.622 **	0.043
20. Isolation																				1	0.370 **	0.864 **
21. Mindfulness																					1	0.379 **
22. Over-identification																						1

* *p* < 0.05; ** *p* < 0.001; **bold** = statistically significant correlations

**Table 4:** Hierarchical Multiple Linear Regression: predictors of the Psoriasis Disability Index (Final Model).

Model	*R*	*R* ^2^	*R*^2^ Adjusted	Standardized Error of the Estimate	*R*^2^ Change	*F* Change	*df*1	*df*2	Significant *F* Change
1	0.168	0.028	0.015	7.270	0.028	2.116	1	73	0.150
2	0.721	0.520	0.470	5.332	0.492	11.454	6	67	<0.001
3	0.835	0.698	0.651	4.328	0.178	12.558	3	64	<0.001
			*B*	*Error*	*β*	*t*	*p*		
1	(Constant)		13.012	3.253		−4.001	<0.001		
	Years of education		−0.929	0.639	−0.168	−1.455	0.150		
2	(Constant)		−2.256	4.275		−0.528	0.599		
	Years of education		−0.889	0.474	−0.160	−1.874	0.065		
	Other diseases		−0.146	1.303	−0.010	−0.112	0.911		
	Family history of psoriasis		1.790	1.271	0.122	1.408	0.164		
	Disease severity perception		−1.267	1.104	−0.112	−1.148	0.255		
	Satisfaction with treatment		−0.175	1.369	−0.011	−0.128	0.899		
	Anxiolytics/antidepressants		1.307	1.491	0.079	0.877	0.384		
	Impact in social life		6.182	0.830	0.719	7.452	<0.001		
3	(Constant)		−0.613	7.159		−0.086	0.932		
	Years of education		−0.897	0.393	−0.162	−2.282	**0.026**		
	Other diseases		−1.590	1.102	−0.108	−1.443	0.154		
	Family history of psoriasis		0.958	1.089	0.065	0.880	0.382		
	Disease severity perception		−0.963	0.903	−0.085	−1.067	0.290		
	Satisfaction with treatment		0.984	1.144	0.062	0.861	0.393		
	Anxiolytics/antidepressants		0.318	1.240	0.019	0.257	0.798		
	Impact in social life		3.160	0.886	0.367	3.567	**0.001**		
	Body image (total)		0.228	0.062	0.433	3.694	**<0.001**		
	Acceptance Action (total)		0.119	0.083	0.134	1.444	0.153		
	Self-compassion (total)		−0.039	0.048	−0.083	−0.799	0.427		

Notes: *R* = multiple correlation coefficient; *R*^2^ = explanation factor (how much of the variation is explained by the model); *R*^2^ adjusted = percentage of variance that would not be explained by the model if it were derived from the population instead of a sample; *df*1 = numerator degrees of freedom; *df*2 = denominator degrees of freedom; *β* = slope gradient; *F* = analysis of variance ANOVA; *t* = informs whether the value of *β* is different from 0; *p* = significance value; **bold** = statistically significant differences.

**Table 5:** Path analysis of body image as a mediator of acceptance and disease severity perception, and the Psoriasis Disability Index; indirect effects.

Independent Variable	Mediator Variable	Dependent Variable	*B* Mean Indirect Effect	*SE* of Mean	95% *CI* Mean Indirect Effect (Lower and Upper)	*p*
Disease severity perception	Body image	Psoriasis disability index	0.035	0.004	0.028; 0.043	<0.001 ***
Acceptance and action			0.444	0.041	0.368; 0.530	<0.001 ***

*** *p* < 0.001; *B* = the indirect effect represents the portion of the relationship between *X* (independent variable) and *Y* (dependent variable) that is mediated by body image; *SE* = Standard error; *CI* = confidence interval.
